# Laser Phototherapy Enhances Mesenchymal Stem Cells Survival in Response to the Dental Adhesives

**DOI:** 10.1155/2015/671789

**Published:** 2015-03-23

**Authors:** Ivana Márcia Alves Diniz, Adriana Bona Matos, Márcia Martins Marques

**Affiliations:** Restorative Dentistry Department, School of Dentistry, Universidade de São Paulo, Avenida Professor Lineu Prestes 2227, 05508-000 Cidade Universitária, SP, Brazil

## Abstract

*Background*. We investigated the influence of laser phototherapy (LPT) on the survival of human mesenchymal stem cells (MSCs) submitted to substances leached from dental adhesives. *Method*. MSCs were isolated and characterized. Oral mucosa fibroblasts and osteoblast-like cells were used as comparative controls. Cultured medium conditioned with two adhesive systems was applied to the cultures. Cell monolayers were exposed or not to LPT. Laser irradiations were performed using a red laser (GaAlAs, 780 nm, 0.04 cm^2^, 40 mW, 1 W/cm^2^, 0.4 J, 10 seconds, 1 point, 10 J/cm^2^). After 24 h, cell viability was assessed by the 3-[4,5-dimethylthiazol-2-yl]-2,5-diphenyl tetrazolium bromide reduction assay. Data were statistically compared by ANOVA followed by Tukey's test (*P* < 0.05). *Results*. Different cell types showed different viabilities in response to the same materials. Substances leached from adhesives were less cytotoxic to MSCs than to other cell types. Substances leached from Clearfil SE Bond were highly cytotoxic to all cell types tested, except to the MSCs when applied polymerized and in association with LPT. LPT was unable to significantly increase the cell viability of fibroblasts and osteoblast-like cells submitted to the dental adhesives. *Conclusion*. LPT enhances mesenchymal stem cells survival in response to substances leached from dental adhesives.

## 1. Introduction

Laser phototherapy (LPT) is a therapeutic approach that promotes healing or repair of injured tissues. For this reason, LPT has been used as an adjuvant therapy in various clinical procedures in dentistry. In fact, LPT has been proven effective in improving dental tissue repair when applied to the dental tissues, such as after cavity preparation and restoration [1–3]. In a previous work, our group has observed that LPT was able to increase cell viability of cultures exposed to substances released from the dental bleaching gels [4]. However, this study was performed using dental pulp cells in an advanced stage of differentiation. In the dental pulp tissue, mesenchymal stem cells (MSCs) are known to play an important role during dental pulp tissue healing or repair. In fact, the dental pulp holds cells in multiple stages of commitment, which, therefore, may interplay for the tissue homeostasis [5, 6]. Unlike end-stage cells, MSCs can undergo asymmetric division, that is, one cell differentiates toward a differentiated cell, while the other replicates into another mesenchymal cell [7]. Accordingly, MSCs have the ability of self-renewal and are able to differentiate into at least two cell types [8]. LPT has already shown improvement in the MSCs proliferative rate and differentiation [9–11]. Current results stress that the association of laser and MSCs may be of particular relevance in the dentistry field.

Dental adhesives are materials commonly applied to the dental substrates and may lead to a certain degree of cytotoxicity in cell cultures. The percentage of unconverted resin monomers leads to the risk of formation of oxygen-free radicals (ROS), which, in turn, may result in inflammation and postoperative sensitivity [12–14]. Due to incomplete polymerization, uncured monomers are able to percolate dentinal tubules and reach dental pulp tissue [12]. Regarding the relevance and plasticity properties of MSCs, it is of interest to verify whether LPT could help MSCs overwhelm noxious substances derived from these materials. Bearing this in mind, the aim of this study was to test the effect of LPT on the survival of MSCs using substances leached from dental adhesives as a model of cytotoxicity.

## 2. Methods

This study was previously approved by the Research Ethics Committee of the School of Dentistry of the University of São Paulo (CAE: 03511012.5.0000.0075).

### 2.1. Cell Culture

**Figure 1:**
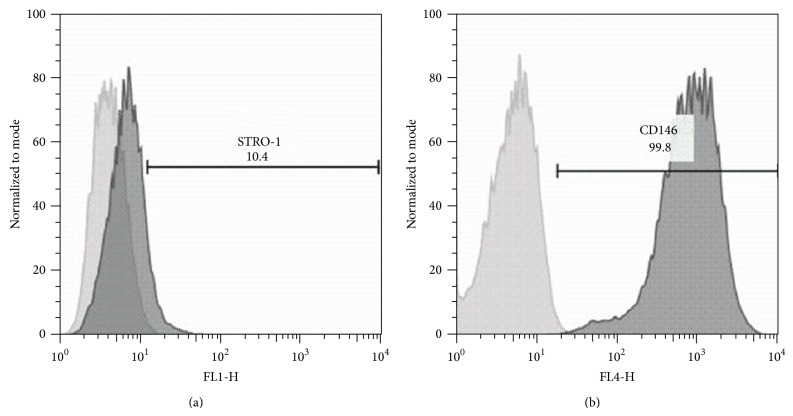
Characterization of the stem cells from human exfoliated deciduous teeth through the expression profile of mesenchymal stem cell markers: STRO-1 (a) and CD146 (b). Observe the positivity to both markers.

Mesenchymal stem cells (MSCs) derived from human exfoliated deciduous teeth were isolated according to Miura et al. [15] and characterized as showing positivity for mesenchymal stem cell surface markers (STRO-1 and CD146) (Figure 1). Other cell types studied were fibroblasts of oral mucosa and osteoblast-like cells. The cell lineages were kindly provided by the Basic Research Laboratory at the School of Dentistry of the Universidade de São Paulo.

Aliquots of the cultures were thawed and grown as follows: MSCs were grown in DMEM-HAM's F12 (LGC Biotechnology, Cotia, Brazil) and supplemented with 20% fetal bovine serum (Hyclone, Logan, USA), 1% L-glutamine, 1% nonessential amino acids, and 1% penicillin/streptomycin. Fibroblasts and osteoblast-like cells were grown in high-glucose DMEM and supplemented with 15% fetal bovine serum and 1% solution of penicillin, streptomycin, and amphotericin B. All cell types were maintained in an incubator at 37°C, in a humidified atmosphere containing 5% CO_2_ and 95% air.

### 2.2. Substances

Two types of dental adhesives were used as follows: (1) etch and rinse (Adper Single Bond 2, 3 M ESPE, St. Paul, USA) and (2) self-etch (Clearfil SE Bond, Kuraray Co., Osaka, Japan), described in detail in Table 1.

### 2.3. Conditioned Medium

A culture medium conditioned by the dental adhesives was used to reproduce the substances leached from the Adper Single Bond 2 or Clearfil SE Bond [16]. The conditioned medium was obtained as follows: using a microbrush tip, one drop of each material was dispensed to a predelineated area at the bottom of 1.5 mL microtubes. The Clearfil SE Bond comes with primer and adhesive components in separate vials. Considering this, the bond and the primer were applied with the primer on the top to simulate the clinical situation where the primer remains closer to the dentin than the bond. Next, 1 mL of culture medium was added to the microtubes containing each material. These microtubes were kept at 37°C for 1 h.

Photopolymerization, when applied, was performed using a light emitting diode (Elipar free light LED curing light, 3 M ESPE) and previously checked with a radiometer, in accordance with the manufacturer's instructions for each adhesive system.

### 2.4. Laser Phototherapy (LPT)

Laser phototherapy was performed using a continuous wave gallium-aluminum-arsenide (GaAlAs, 780 nm) diode laser (Twin Flex II, MMOptics, São Carlos, Brazil) with a spot size of 0.04 cm^2^. The irradiations were performed in contact and punctual mode, with the following parameters: output power of 40 mW, power density of 1 W/cm^2^, energy of 0.4 J, and energy density of 10 J/cm^2^. In order to avoid indirect light exposure wells adjacent to the test well were empty. Each well was irradiated once in a central point for 10 s. Laser parameters were chosen based on a previous study [4]. The output power was checked with a power meter (Lasercheck, Coherent Inc., Santa Clara, USA), before and after the irradiations.

### 2.5. Experimental Groups

The experimental groups are presented in Table 2.

### 2.6. Experiments

Each cell type was seeded at a cell density of 1 × 10^4^ cells/well in quadruplicate into 96 microtitration well-plates. Twenty-four hours later, the culture medium was replaced by the conditioned medium. Next, the cultures were either submitted or not to LPT, according to the specific experimental group studied. The conditioned medium was left in contact with the cells for 1 hour and then replaced by fresh medium. The plates were incubated for another 24 hours and then subjected to the cell viability assay.

### 2.7. Cell Viability Assay (MTT)

Analysis of cell viability was based on the measurement of mitochondrial activity using the 3-[4,5-dimethylthiazol-2-yl]-2,5-diphenyl tetrazolium bromide reduction assay (Vybrant MTT Cell Proliferation Assay Kit, Invitrogen, Carlsbad, USA), according to the manufacturer's instructions. Immediately following the end of the test procedures, the optical density was read in a spectrophotometer (Biotek II Biochrom Ltd., Eugendorf, Austria) using a 562 nm filter. The mean optical density of the positive control group was considered as 100%.

### 2.8. Statistical Analysis

Each experiment with four replicates per group was repeated three times. Data were compared by ANOVA followed by Tukey's test using the GraphPad Prism 5.0 software (GraphPad Software Inc., La Jolla, USA). The level of significance was 5% (*P* ≤ 0.05).

## 3. Results

**Figure 2:**
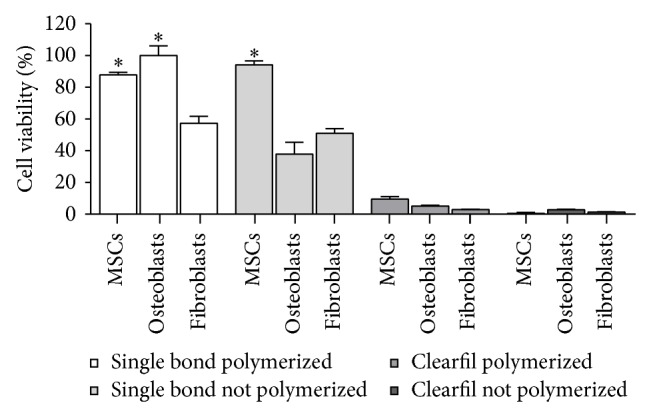
Graphic representation of the cell viability of all cell lineages (MSCs, fibroblasts, and osteoblastic-like cells) in response to substances leached from the dental adhesives: Adper Single Bond 2 and Clearfil SE Bond, whether or not polymerized. ^*^Significantly higher than all other groups (*P* < 0.05).

The cell viabilities of the different cell types in response to the substances leached from the polymerized and nonpolymerized dental adhesives are graphically represented in Figure 2. Different cell types responded differently to the same material. The highest cell viabilities were observed in response to substances leached from Adper Single Bond 2, especially when polymerized.

MSCs and osteoblast-like cell cultures submitted to substances leached from polymerized Adper Single Bond 2 presented similar cell viabilities (*P* > 0.05), which were significantly higher than those of fibroblast cultures (*P* < 0.05) (Figure 2). MSCs submitted to substances leached from nonpolymerized Adper Single Bond 2 presented cell viabilities significantly higher than those of the other cell types (*P* < 0.05). Substances leached from the Clearfil SE Bond, whether polymerized or nonpolymerized, caused a high percentage of cell death in all cell types tested (Figure 2).

**Figure 3:**
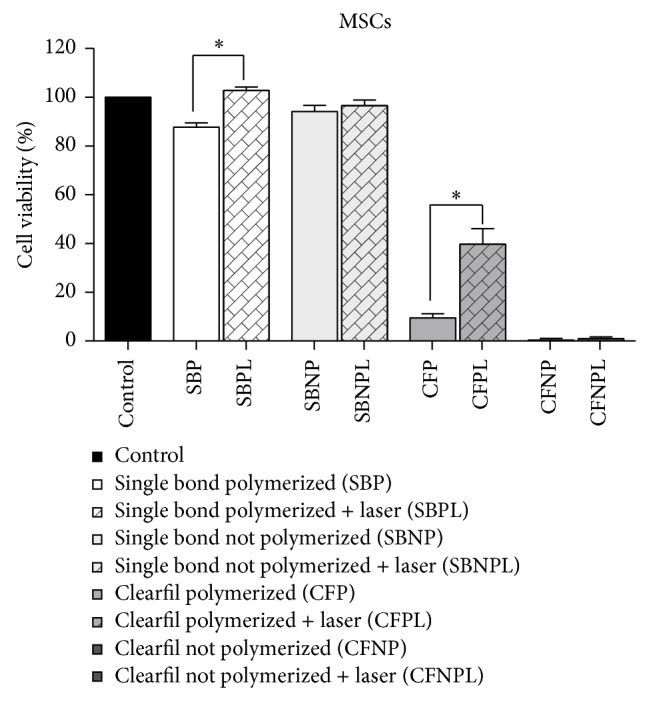
Graphic representation of the cell viabilities of MSCs in response to the substances leached from the dental adhesives: Adper Single Bond 2 and Clearfil SE Bond, whether or not polymerized and followed by LPT or not. ^*^Significantly different from the nonirradiated group submitted to the same adhesive system.

**Figure 4:**
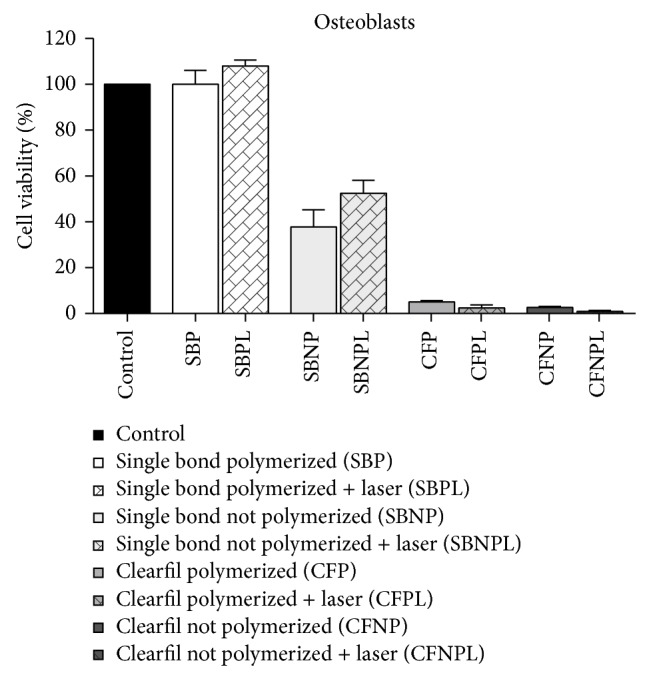
Graphic representation of the cell viabilities of osteoblast-like cells in response to the substances leached from the dental adhesives: Adper Single Bond 2 and Clearfil SE Bond, whether or not polymerized, followed by LPT or not. There are no differences between irradiated and nonirradiated cells submitted to the same adhesive system.

**Figure 5:**
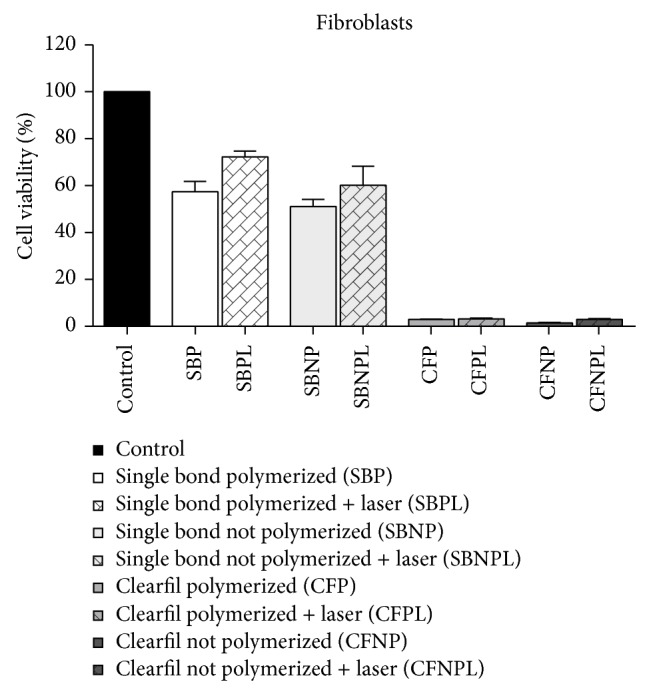
Graphic representation of the cell viabilities of fibroblasts in response to the substances leached from the dental adhesives: Adper Single Bond 2 and Clearfil SE Bond, whether or not polymerized and followed by LPT or not. There are no differences between irradiated and nonirradiated cells submitted to the same adhesive system.

The LPT effects on the cell lineages are graphically represented in Figures 3 to 5. The cell viabilities of MSCs submitted to the substances leached from all the materials, followed by LPT, were higher than or at least similar to those of the nonirradiated cultures submitted to the same conditioned medium. MSCs treated by LPT presented significantly higher cell viabilities when submitted to both polymerized adhesives tested (*P* > 0.05) (Figure 3). The cell viabilities of osteoblast-like cells (Figure 4) and fibroblasts (Figure 5) submitted to the substances leached from all the materials followed by LPT were similar to those of the nonirradiated cultures submitted to the same conditioned medium (*P* > 0.05).

## 4. Discussion

The role of MSCs in response to damaged odontoblasts due to cavity preparation [17] has drawn attention to the response of these cells facing other injuries, such as dental materials percolation through the dentinal tubules [14, 18–21]. Based on the above, our hypothesis was that LPT could improve the survival of MSCs subjected to noxious substances derived from the dental materials. To verify this hypothesis, prior to LPT, two types of dental adhesives were used to imbalance the ideal culture conditions for MSCs, fibroblasts, and osteoblasts-like cells. We found that LPT significantly improved survival of MSCs. In spite of that, overall, no increased cell survival was observed for fibroblast or osteoblast-like cells.

In this study, culture media conditioned by the dental adhesives were used as harm stimuli to test the LPT biostimulation. In the tested conditions, Clearfil SE Bond was highly cytotoxic to all cell lines tested, regardless of being polymerized or not. Substances leached from Adpter Single Bond, regardless of being polymerized or not, were less cytotoxic to MSCs than to the other cell types. Overall, MSCs were less sensitive to toxic substances released by the adhesives, compared to the other cell types tested. These results may be partially explained by the aforementioned properties of MSCs. Their high proliferative nature and plasticity [15] may have contributed to their better response to the noxious substances. In fact, MSCs are involved in the reparative mechanisms of the dental pulp [7] and are recruited to replenish lost specialized cells, such as odontoblasts. In contrast, oral mucosa fibroblasts and osteoblast-like cells are more demanding as end-stage cells and may not respond to stressful conditions at the same level of undifferentiated cells [22].

LPT was able to improve the survival of MSCs to the cytotoxic effect of both adhesive systems when applied after polymerization. On the other hand, for fibroblasts and osteoblast-like cell lineages, LPT was not able to significantly offset the cytotoxic effects of substances released from dental adhesives. Additionally, it was observed that when the materials promoted slight cytotoxicity in the cell lines, LPT had a minimal influence on the improvement of cell survival. In fact, LPT seems to act mainly on cells with compromised cellular functions [23–25]. This can be confirmed by the results obtained for MSCs in the Clearfil SE Bond polymerized groups, whether irradiated or not, which showed to be highly cytotoxic. The percentage of cell viability was very low when the material was applied to the cell cultures, but after irradiation, cell survival rates increased significantly. In other cell types, and in the groups that presented moderate cytotoxicity, although LPT did not significantly increase cell viability, a trend toward improved cellular response could be observed.

With wavelengths in the red or near-infrared ranges, the energy emitted by the laser is capable of being absorbed by cellular components resulting in modulatory effects on basic cellular functions, especially in tissues subjected to stress conditions [26]. Although we cannot mechanistically explain our current results, some previous studies corroborate to elucidate the positive effect of LPT observed here. Laser irradiation can upregulate levels of mRNA of Notch-1, which play an important role in MSCs self-renewal [9]. Accordingly, the modulation of channel gating by laser light may be a critical step in the upregulation of Notch-1 signaling in MSCs, thus stimulating their proliferation [9]. Another study reported that laser irradiation is able to inhibit NF-*κ*B nuclear translocation due to LPS stimulation through an increase in the intracellular level of cyclic AMP (cAMP), suppressing, and, therefore, the release of important proinflammatory cytokines (COX-2, IL-1B, IL-6, and IL-8) [27]. As such, both studies suggest that LPT can help MSCs overwhelm biological stressful situations.

Overall, the Clearfil SE Bond showed severe cytotoxicity to all cell types, whereas Adper Single Bond 2 was reasonably well tolerated. These results are consistent with others described in the literature, although different experimental conditions were reported [14, 18, 28, 29]. Demirci et al. [14] found that Clearfil SE Bond leads to decreased cell viability in a concentration-dependent mode. In fact, dental adhesives cause an imbalance in the cellular redox state with the generation of reactive species of oxygen in cultured dental pulp cells. The ROS formation can interfere with the signal transduction regulating cell survival pathways [30, 31].

The higher cytotoxicity of Clearfil SE Bond in relation to Adper Single Bond 2 can be partially explained by the pH composition. Adper Single Bond 2 is a total etch adhesive and has no acidic monomers in its composition. On the other hand, Clearfil SE Bond is a self-etch adhesive and thus has acidic agents incorporated into the resinous materials, leading to a pH of about 2. Therefore, immediately after the adhesive system came into contact with the culture medium, there was a change in color from orange to yellow; and the yellow remained until the system was applied on the cell cultures. This means that the pH was very low and the buffering capacity of the culture medium was not enough to neutralize the acidic substances leached from the Clearfil SE Bond. Apart from it, this is an* in vitro* study conducted on cultured cells. As such, it has limitations and does not represent the* in vivo* physiology of the dental pulp tissue.

## 5. Conclusions

In summary, this preliminary data suggest that LPT is able to modulate cellular functions to improve MSCs viability under harm stimulus produced* in vitro*. Further studies should be conducted to verify the mechanism of action of LPT in these cells. Under the limit conditions of this study it was concluded that LPT is able to enhance the survival of mesenchymal stem cells after contact to substances leached from dental adhesives.

## Figures and Tables

**Table 1:** Adhesives tested.

Name, brand (lot number)	Class	System	Composition
Adper Single Bond 2 Adhesive, 3 M ESPE (Lot N200625BR)	Etch and Rinse	1 bottle	Bisphenol A glycidyl methacrylate (Bis-GMA), 2-hydroxyethyl methacrylate (HEMA), dimethacrylates, camphorquinone (CQ), polyacrylic acid, poly (itaconic acid), ethanol, and water

Clearfil SE Bond, Kuraray (Lot 01657A/01108A)	Self-etch	2 bottles	**Primer**: 10-methacryloyloxydecyl dihydrogen phosphate (MDP), HEMA, hydrophilic dimethacrylate, dicamphorquinone, N,N-diethanol-p-toluidine, water **Bond**: MDP, Bis GMA, HEMA, hydrophobic dimethacrylate, dicamphorquinone, N,N-diethanol-p-toluidine, silanized colloidal silica

**Table 2:** Experimental groups.

Groups	Polymerization	Nonirradiated	Irradiated
Cells with no treatment	—	Control	—
Adper Single Bond 2	No	SBNP	SBNPL
Adper Single Bond 2	Yes	SBP	SBPL
Clearfil SE Bond	No	CFNP	CFNPL
Clearfil SE Bond	Yes	CFP	CFPL
